# Longitudinal associations between depressive symptoms and cell deformability: do glucocorticoids play a role?

**DOI:** 10.1007/s00406-024-01902-z

**Published:** 2024-09-16

**Authors:** Julian Eder, Martin Kräter, Clemens Kirschbaum, Wei Gao, Magdalena Wekenborg, Marlene Penz, Nicole Rothe, Jochen Guck, Lucas Daniel Wittwer, Andreas Walther

**Affiliations:** 1 Biopsychology, Faculty of Psychology, TUD Dresden University of Technology, Dresden, Germany; 2 Center for Molecular and Cellular Bioengineering, Biotechnology Center, TUD Dresden University of Technology, Dresden, Germany; 3 Max Planck Institute for the Science of Light & Max-Planck-Zentrum für Physik und Medizin, Erlangen, Germany; 4 School of Psychology, Nanjing Normal University, Nanjing, China; 5 Else Kröner Fresenius Center of Digital Health, Faculty of Medicine and University Hospital Carl Gustav Carus, TUD Dresden University of Technology, Dresden, Germany; 6 Institute of Psychology, Johannes Kepler Universität Linz, Linz, Austria; 7 Institut für Numerische Mathematik und Optimierung, Technische Universität Freiberg, 09599 Freiberg, Germany; 8 Clinical Psychology and Psychotherapy, University of Zurich, Binzmühlestrasse 14, Zurich, 8050 Switzerland

**Keywords:** Depressive symptoms, Cell deformability, Real-time deformability cytometry, Hair cortisol, Hair cortisone

## Abstract

Cell deformability of all major blood cell types is increased in depressive disorders (DD). Furthermore, impaired glucocorticoid secretion is associated with DD, as well as depressive symptoms in general and known to alter cell mechanical properties. Nevertheless, there are no longitudinal studies examining accumulated glucocorticoid output and depressive symptoms regarding cell deformability. The aim of the present study was to investigate, whether depressive symptoms predict cell deformability one year later and whether accumulated hair glucocorticoids mediate this relationship. In 136 individuals (*n*_*female*_ = 100; *M*_*age*_ = 46.72, *SD* = 11.28; age range = 20–65), depressive symptoms (PHQ-9) and hair glucocorticoids (cortisol and cortisone) were measured at time point one (T1), while one year later (T2) both depressive symptoms and hair glucocorticoids were reassessed. Additionally, cell deformability of peripheral blood cells was assessed at T2. Depression severity at T1 predicted higher cell deformability in monocytes and lymphocytes at T2. Accumulated hair cortisol and cortisone concentrations from T1 and T2 were not associated with higher cell deformability and further did not mediate the relationship between depressive symptoms and cell deformability. Elevated depressive symptomatology in a population based sample is longitudinally associated with higher immune cell deformability, while long-term integrated glucocorticoid levels seem not to be implicated in the underlying mechanism.

**Supplementary Information:**

The online version contains supplementary material available at 10.1007/s00406-024-01902-z.

## Introduction

Of all mental disorders, depressive disorders (DD), comprising major depressive disorder (MDD) and persistent depressive disorder (PDD), cause the greatest disease burden and disability worldwide [1], being top-ranked among an exhaustive number of fatal and non-fatal diseases causing disability-adjusted life-years in all ages [2]. MDD is, according to the classification system DSM-5 [3] characterized by a period of at least two weeks of depressive mood or anhedonia in combination with other symptoms such as appetite changes, insomnia/hypersomnia, increased fatigue, or feelings of worthlessness. PDD (formerly dysthymia) is characterized on the basis of depressed mood over a two-year period in combination with symptoms similar to MDD, although PDD is often perceived as less severe in terms of intensity [3]. Nevertheless, both conditions cause severe functional impairment and there are major difficulties in reducing the high prevalence rates [4, 5] and improving DD treatment [6, 7], with up to 30% of patients not achieving remission despite multiple treatment attempts [8–10]. Often, the poor therapeutic efficacy is attributed to the still insufficiently understood pathophysiology of DD [11–14].

There is accumulating evidence of key pathophysiological processes in depression and its etiology occurring directly at the cellular level and that DD and depressive symptoms can impair peripheral blood cell function via disturbed glucocorticoid secretion by the hypothalamus-pituitary-adrenal (HPA) axis or via inflammation [15–21]. Blood cells fulfill multiple functions from primary immune response, over metabolite transport to overall blood flow. Real-time deformability cytometry (RT-DC) represents a new method which enables the investigation of physical properties of blood cells (e.g., cell mechanical features, cell size), thereby providing unprecedented insights into overall blood cell function [22, 23]. Previous research indicates that the assessment of the mechanical state of blood cells, measured by cell deformability, is appropriate to detect and classify human diseases conditions such as spherocytosis, malaria, or COVID-19 infections [24, 25] and is predictive for immune cell activation [26, 27].

Only recently, using RT-DC, we were able to show that in DD and especially in PDD immune cell deformability is increased as compared to healthy controls [28]. Although all major immune cells tended to be more deformable, lymphocytes, monocytes and neutrophils were particularly affected indicating immune cell mechanical changes to occur in DD. This is potentially related to disturbed HPA axis function or dysfunctional immune response, both significantly grounded as prominent biological findings in MDD [18] affecting the cell architecture [29, 30]. In addition, optical traps [31], atomic force microscopy [23, 32], and micropipette aspiration [33] have been used in proof-of-concept studies to reveal mechanical changes in immune cells under pathological conditions. Nevertheless, due to the general dominance of erythrocytes in blood and the consequently increased statistical power to detect associations with cell morphological or mechanical changes, previous studies of interest investigated only the relationship between erythrocyte deformability and a developmental disorder as well as a multi-systemic disease with fatigue as leading symptom [34, 35]. The study by Jasenovec et al. (2019) examined children within the autism spectrum identifying those children with more severe symptoms to show impaired erythrocyte deformability. Further, Saha et al. (2019) reported patients with chronic fatigue syndrome / myalgic encephalomyelitis to exhibit lower erythrocyte deformability than healthy controls. It should be noted here, however, that the current gold standard for measuring cell deformability, namely RT-DC [22, 36–39], was only used in our landmark study comparing individuals with DD and healthy controls [28].

There is a large body of literature examining the relationship between impaired glucocorticoid secretion by the HPA axis, DD [14] and depressive symptoms [15]. Indeed, for some time, it seemed to be widely accepted that MDD and depression severity are associated with increased HPA activity and increased basal cortisol concentrations in the circulation [13, 40]. However, due to the inconclusive findings on cortisol stress reactivity in MDD, this is no longer definitive [14, 41, 42]. Furthermore, investigating new tissue sources such as hair showing mixed associations with regard to DD highlight on the one hand the relevance of the investigation of chronic glucocorticoid secretion over longer time periods [14, 43–47], but on the other hand, it emerges as increasingly important to investigate the effects of impaired chronic glucocorticoid secretion on cell function. Studies increasingly show that the extracellular microenvironment is critical to modify cellular physiology, which ultimately determines cell functionality [48]. Therefore, understanding the interactions between immune cells and DD-related alterations in glucocorticoid secretion appears to be increasingly important for understanding differences in immune cell deformability in health and disease.

In addition to increased cortisol levels and the often identified chronic low-grade inflammation in individuals suffering from DD [18], Lynall et al. (2020) further reported elevated numbers of immune cells in screened depressed individuals compared to controls [49]. It is known that increased levels of glucocorticoids and catecholamines lead to increased immune cell count, as cells demarginate from the vessel walls. Interestingly, these observations were associated with cellular softening [29]. It is suggested that the underlying mechanism leading to increased immune cell counts in depressed individuals is rooted in the effect of elevated glucocorticoid levels remodeling the actin cytoskeleton of blood cells and thereby softening leukocytes and enabling them to demarginate from the vessel wall [29]. Not only starts the actin cytoskeleton to be remodeled with continuously elevated levels of glucocorticoids, but also are lipid metabolism and composition crucially affected resulting in increased softening and bending of blood cells [20, 33, 50–52]. This cascade of processes ultimately impairs blood cell function and may be an underlying cause of symptoms of fatigue and exhaustion in DD. The disturbed immune cell function in connection with subsequent dysregulated cytokine release could trigger sickness behavior with anhedonia and fatigue [53].

Therefore, we hypothesize that high levels of depressive symptoms are longitudinally associated with increased immune cell deformability. We further hypothesize that continuously high glucocorticoid levels over one year are associated with elevated immune cell deformability and mediate the relationship between depressive symptoms and immune cell deformability.

## Method

### Study design

This observational study used a longitudinal design with a baseline measurement and a one-year follow-up measurement. Cross-sectional results of the follow-up study are reported elsewhere [28]. Baseline examination of participants took place between October and December 2018, while follow-up examinations were conducted approximately one year later. The follow-up study, entitled *Mood-related Morpho-rheological Changes in Peripheral Blood Cells* (Mood-Morph), used a participant pool of a large-scale prospective cohort study to identify eligible participants and obtain pre-assessed baseline data (study details of the cohort study are provided elsewhere [54]). Eligible participants were asked via e-mail whether they were willing to participate in an add-on study to the prospective longitudinal study.

For the study, participants with elevated depressive symptoms, as measured by the German version of the Patient Health Questionnaire (PHQ-9 [55]), and gender- and age-matched healthy controls were identified from the participant pool. These participants provided for the baseline measurement self-report data on (T1) sociodemographic information, depressive symptomatology and further health-related factors. Also, collected hair samples for glucocorticoid quantification were obtained at T1. At the follow-up time point (T2), the psychometric assessment and hair sampling was repeated. In addition, blood samples were obtained to measure cell deformability. The study was approved by the local ethics committee of the Dresden University of Technology (EK182042019) and all participants gave written informed consent to participate in the study. For study expenses, participants received cash or a cinema voucher worth 15 € in T1 and 15 € in T2.

### Participants

Study inclusion was a participants’ age between 18 and 68 years and sufficient German language skills. Furthermore, in terms of depression levels measured with the PHQ-9, only subjects with a T1 PHQ-9 score > 10 (high risk of depression) as well as age- and sex-matched subjects with a T1 PHQ-9 score < 5 (low risk of depression) were included. The aim of this procedure was to obtain a sample of participants with high and low depressive symptoms at baseline and to be able to identify change in depressive symptoms over one year. Interested study participants were excluded if they had any kind of blood disorder that could affect blood cell deformability. Potential participants who were contacted to participate in the Mood-Morph study (the detailed sample recruitment can be taken from Walther et al. (2022)), were informed in the study invitation and informational email that certain diseases, such as spherocytosis and blood-related diseases in general, precluded participation [25]. Potential participants with a cold or other acute infections no longer than two weeks ago were not included to exclude acutely infectious participants. Acute medical conditions such as blood diseases, cancer, severe cardiovascular diseases, or pregnancy led to exclusion, while in the population very prevalent conditions such as hypertension or hypothyroidism did not lead to exclusion. These conditions were recorded and used as covariate in the analyses in form of drug specific categories (e.g., for hypertension—antihypertensive drugs).

### Procedures

Participants provided sociodemographic and health-related information (e.g., gender, age, medical conditions, medication) as well as depressive symptom severity and information on their general health online via the study-homepage platform. By using a personalized code, participants accessed a private study space, signed first consent forms regarding data privacy, clinical data collection and saving, and hair/blood sampling and completed subsequently study questionnaires. For depression severity the PHQ-9 [55] and for general health the Short Form Health Questionnaire (SF-12 [56]) was completed. While the SF-12 measures general physical and psychological health, the PHQ-9 is a nine-item self-report questionnaire for the measurement of depression severity, rating the frequency of DSM-IV / DSM-5 diagnostic criteria for MDD (e.g., feeling down, depressed, or hopeless) during the past two weeks on a four-point Likert scale (0 = not at all, 3 = nearly every day). Scores ≥ 10 were consistently shown to identify with high sensitivity and specificity cases of MDD [57, 58].

To obtain capillary blood and scalp hair samples at T2, participants arrived at the Dresden University of Technology at the Department of Biopsychology, where trained personnel performed blood and hair sampling. After the bio-sampling took place, the depressive episode section of a standardized interview, named the Composite International Diagnostic Interview (DIA-X-5/CIDI) was conducted by trained interviewers [59]. To rule out bipolar disorders, a mania-screening questionnaire, consisting of the initial questions of the DIA-X-5 (hypo-)manic episodes’ section, was conducted to detect any lifetime (hypo-) mania-symptoms. Analyses regarding differences in cell deformability between DD (MDD and PDD) and healthy participants can be taken from Walther et al. (2022) and are not part of this manuscript. Duration of the examination at T2 was approximately one hour.

### Real-time deformability cytometry (RT-DC)

A 20 µl capillary blood sample was extracted from the fingertip of participants using a safety lancet (Safety-Lancet Normal 21, Sarstedt, Nümbrecht, Germany) and harvested in a capillary (Minivette POCT, 20 µl, Sarstedt, Nümbrecht, Germany). The blood was immediately resuspended in 380 µl RT-DC measurement buffer containing 0.6% methylcellulose in phosphate buffered saline (CellCarrierB, Zellmechanik Dresden, Germany) in a microcentrifuge tube. Blood-samples were then transferred to the Department of Cellular Machines at the Biotechnology Center of the Dresden University of Technology, where the samples were measured using an RT-DC device. Maintained at room temperature, samples were measured within three hours since sampling according to a protocol published elsewhere [25].

In brief, blood was flushed through a microfluidic channel constriction 20 μm x 20 μm in cross section (Flic20, Zellmechanik Dresden, Germany) by applying a constant flow rate. An image of every measured blood cell was taken by a high-speed camera and cell deformability and cell size were calculated [22]. RT-DC measurements were controlled by the acquisition software Shape-In2 (Zellmechanik Dresden, Germany). The different blood cell types were classified by utilizing artificial intelligence-based image classification as published elsewhere [36] and mean values for cell deformability and cell size of every donor and blood cell type were extracted. Subsequently, we only focus on immune cells and erythrocytes.

### Hair sampling and liquid chromatography and mass-spectrometry (LC-MS)

Three hair strands (each containing at least 20 mg of hair) were cut as close as possible to the scalp from the posterior vertex for quantification of hair cortisol and cortisone. Hair sample processing and analysis were performed as described in our published protocols for glucocorticoids [60, 61]. Preprocessing of the samples and subsequent biochemical analysis were performed by Dresden LabService GmbH (Tatzberg 47, 01307, Dresden, Germany). Samples with a minimum hair length of 3 cm were cut into 3 cm segments from the scalp site representing the integrated hormone concentration over the last three months (e.g., average hair growth rate of 1 cm per month [62]) and weighed into 7.5 mg of whole, non-pulverized hair and then washed with 2.5 ml of isopropanol according to the protocol of Gao et al. (2013). Biochemical analysis was performed by liquid chromatography coupled with tandem mass spectrometry (LC-MS/MS) as described in detail elsewhere [61]. The intra- and inter-assay coefficients of variation for cortisol and cortisone are less than 8.8% and 8.5%, respectively. The lower limit of detection for cortisol and cortisone is 0.09 pg/mg.

### Statistical analysis

Statistical analyses were run in R v4.3.0 (RStudio v. 2023.06.0 [63]). The longitudinal association between depressive symptoms at T1 and cell deformability at T2 (lymphocytes, monocytes, neutrophils, granulo-monocytes [cluster of monocytes and all cell types of granulocytes] and erythrocytes) was examined by hierarchical regression analyses with bootstrapping (*R* = 1000) and robust standard errors (HC4) to obtain robust effect estimations for the entire sample (*n* = 136). Data was checked for linearity, normal distribution of the residuals, heteroscedasticity, multicollinearity and outliers. Hierarchical regression analyses were conducted in two steps: First, age, BMI, gender, and psychopharmaceutical intake, measured at T2, were included as confounders. Second, with respect to the analyses for the entire sample, depressive symptoms at T1 were included in the regression model to account for additionally explained variance. Additionally, we run a mediation analyses with the process macro, provided by Hayes (2022) [64], to examine the mediating effect of accumulated hair glucocorticoids (cortisol and cortisone at T1 + T2, respectively) between depressive symptoms (PHQ-9 at T1) and cell deformability at T2. *P*-values were adjusted using Holm-Bonferroni correction for multiple comparisons [65] by the number of cell types. Cortisol and cortisone data from both measurements were log-transformed due to their non-normal distribution. All hypotheses were tested two-tailed in regard to cell deformability. Pearson and Kendall correlation coefficients [66] were tested to check for possible (non-)linearity relationships between depressive symptom severity and cell area as well as depressive symptom severity or glucocorticoids and cell count, obtained by the classification algorithm [36]. ANOVA was used to check for any differences in medication intake groups and cell deformability for each cell type.

## Results

### Sample description

In total, 136 (*n*_*female*_ = 100) individuals, who completed the PHQ-9 at T1 and T2 were used for subsequent analyses investigating the longitudinal association between depressive symptoms, glucocorticoid secretion and immune cell deformability. Sample characteristics of the entire sample are presented in Table 1. Moreover, hair segments could not be obtained from all subjects due to lack of sufficient hair of some participants. Regarding cortisol analyses, one outlier was additionally excluded due to a hair cortisol concentration above 100 pg/mg. Regarding cortisol output *n* = 113 observations were available at both time points, while for cortisone output *n* = 115 observations were available at both time points.

**Table 1:** Sample characteristics according to the entire sample

Sample characteristics	Entire sample (*n* = 136)	Range (min – max)
Age M (SD)	46.72 (11.28)	20–65
Sex females (%)	100 (73.53%)	–
Body mass index M (SD)	25.51 (4.88)	17.57–40.09
Psychopharmaceutical intake frequency	23 (16.91%)	–
Medication frequency	74 (54.41%)	–
PHQ-9 T1 M (SD)	8.74 (6.98)	0–25
PHQ-9 T2 M (SD)	7.65 (6.03)	0–25
Hair cortisol T1 (pg/mg) M (SD)^a^	8.84 (6.43)	1.75–37.00
Hair cortisol T2 (pg/mg) M (SD)^a^	9.99 (8.45)	1.00–45.99
Hair cortisol T1 + T2 (pg/mg) M (SD)^a^	18.83 (13.72)	3.80–66.89
Hair cortisone T1 (pg/mg) M (SD)^b^	27.33 (18.50)	4.71–90.39
Hair cortisone T2 (pg/mg) M (SD)^b^	32.34 (23.15)	6.51–93.22
Hair cortisone T1 + T2 (pg/mg) M (SD)^b^	59.67 (39.05)	13.34–169.13

*Note* T1 = time point 1, T2 = time point 2, PHQ-9 = Patient Health Questionnaire 9, *n* = number of participants, *M* = mean, *SD* = standard deviation, ^a^number of hair cortisol observations at T1 and T2 = 113, ^b^number of hair cortisone observations T1 and T2 = 115

### Control of confounders

No significant correlations between depressive symptoms (PHQ-9 T1) and mean cell area size (see Supplementary Table 1) and between depressive symptoms (PHQ-9 T1) and cell count (see Supplementary Table 2) were found for any cell type. Furthermore, accumulated hair cortisol and cortisone levels were not associated with cell count of any cell type, detected by the cell classification algorithm (see Supplementary Tables 3 and 4). Moreover, cell deformability did not differ with respect to the six different medication groups (see Supplementary Text 1).

**Table 2:** Mediating role of glucocorticoids towards the association of depression symptoms and cell deformability

	Outcome Regression Fit				Mediation Paths, ß				Indirect Effects	
	*F* ^*HC4*^	*df*	*p*	*R* ^2^	*a*	*b*	*c*	*c‘*	ß	95% BootCI
**Lymphocytes (with covariates: age, gender, BMI, psychopharmaceutical intake)**										
Cortisol (T1 + T2)	3.327	6,106	0.005	0.15	− 0.07	0.03	0.16^+^	0.16^+^	− 0.00	[-0.04, 0.02]
Cortisone (T1 + T2)	3.279	6,108	0.005	0.15	− 0.07	0.06	0.15^+^	0.16^+^	− 0.00	[-0.04, 0.02]
**Monocytes (with covariates: age**, **gender**, **BMI**, **psychopharmaceutical intake)**										
Cortisol (T1 + T2)	1.762	6,106	0.114	0.08	− 0.07	− 0.06	0.20*	0.20*	0.00	[-0.02, 0.04]
Cortisone (T1 + T2)	1.686	6,108	0.131	0.07	− 0.07	0.03	0.19*	0.20*	− 0.00	[-0.03, 0.02]
**Neutrophils (with covariates: age**, **gender**, **BMI**, **psychopharmaceutical intake)**										
Cortisol (T1 + T2)	0.451	6,106	0.843	0.03	− 0.07	0.08	− 0.02	− 0.01	− 0.01	[-0.05, 0.02]
Cortisone (T1 + T2)	0.699	6,108	0.651	0.04	− 0.07	0.14	− 0.03	− 0.02	− 0.01	[-0.05, 0.02]
**Granulo-monocytes (with covariates: age**, **gender**, **BMI**, **psychopharmaceutical intake)**										
Cortisol (T1 + T2)	0.369	6,106	0.897	0.02	− 0.07	0.04	− 0.02	− 0.02	− 0.00	[-0.04, 0.02]
Cortisone (T1 + T2)	0.573	6,018	0.751	0.03	− 0.07	0.12	− 0.03	− 0.02	− 0.01	[-0.05, 0.02]
**Erythrocytes (with covariates: age**, **gender**, **BMI**, **psychopharmaceutical intake)**										
Cortisol (T1 + T2)	0.814	6,106	0.562	0.06	− 0.07	− 0.04	0.08	0.08	0.00	[-0.02, 0.04]
Cortisone (T1 + T2)	1.131	6,108	0.350	0.06	− 0.07	0.01	0.08	0.08	− 0.00	[-0.03, 0.03]

*Note* Independent variable = PHQ-9 (T1); Dependent variable = Cell deformability (T2). Mediators: accumulated hair cortisol and hair cortisone concentrations (T1 + T2). Model fit for hair cortisol path a: *F*(5,107) = 3.221, *p* = .0095, *R²* = 0.13; Model fit for hair cortisone path a: *F*(5,109) = 4.341, *p* = .001, *R²* = 0.15; 95%-CI is based on 1000 bootstrap replicates. Standard errors and F-values are calculated with HC4. *p < .05; ^+^*p* < .10

### Association between depression severity and cell deformability

**Fig. 1:**
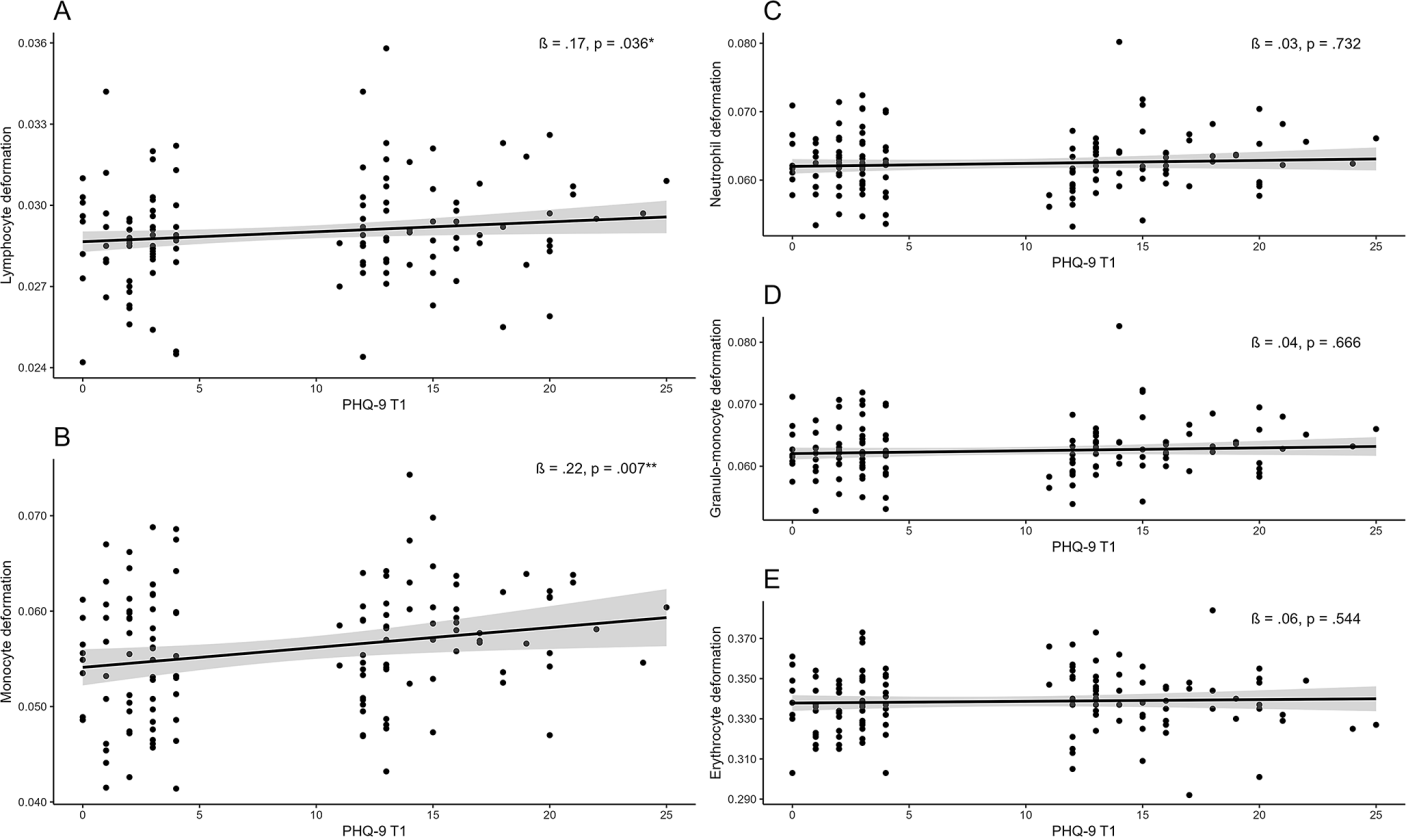
Robust regression analysis regarding the association of depressive symptoms (T1) on cell deformability (T2). Standardized regression coefficients of hierarchical regression analyses are depicted. Age, gender, BMI and psychopharmaceutical intake at T2 were included as confounders

Hierarchical regression analyses with robust standard errors regarding the association of depressive symptom scores at T1 and cell deformability at T2 for each cell type are depicted in Fig. 1 and Supplementary Tables 5–9. Regarding lymphocyte deformability, model 1 and model 2 were significant (M(odel) 1: *F*(4, 131) = 4.495, *p* = .002, *R²* = 0.121, *Adj. R*² = 0.094; M 2: *F*(5,130) = 4.428, *p* < .001, *R²* = 0.146, *Adj. R² =* 0.113, ∆*R²* = 0.025) with BMI (ß = 0.323, *p* = .001, 95% Bca-CI [0.00, 0.00]) and depressive severity at T1 (ß = 0.172; *p* = .036, 95% Bca-CI [0.00, 0.00]) being a significant predictor for lymphocyte cell deformability in model 2. However, depressive severity at T1 did not remain significant, if applied to Holm-Bonferroni correction (*p*_*corrected*_ = 0.144). Model 1 and model 2 did not differ significantly, *F*(1,130) = 3.780, *p* = .054. Normal distribution of residuals in model 1 and 2 (M1: *W* = 0.979, *p* = .038; M2: *W* = 0.981, *p* = .0497) was violated as single assumption.

Ambiguous results were found regarding monocyte deformability: The overall model 1 and model 2 were not significant (M1: *F*(4, 131) = 1.325, *p* = .264, *R²* = 0.039, *Adj. R²* = 0.010; M2: *F*(5,130) = 2.201, *p* = .058, *R²* = 0.078, *Adj. R² =* 0.043, ∆*R²* = 0.039). However, the confounder gender (ß = 0.393, *p* = .042, 95% Bca-CI [0.000, 0.005]) was significant in model 1 both calculated without (*p* = .049) and with robust standard errors (*p* = .042). Moreover, in model 2, both gender (ß = 0.380, *p* = .040, 95% Bca-CI [0.000, 0.005]) and depressive severity at T1 (ß = 0.216; *p* = .007, 95% Bca-CI [0.000, 0.000]) were identified as significant predictors for monocyte deformability. Depressive severity at T1 did survive Holm-Bonferroni correction (*p*_*corrected*_ = 0.035). Moreover, model 1 and model 2 did differ significantly, *F*(1,130) = 5.522, *p* = .020. All assumptions were met. Exploratively, depressive severity at T1 as single predictor in a regression model led to a significant model, *F(*1,134) = 6.786, *p* = .010, *R² =* 0.048, A*dj. R² =* 0.041, ß = 0.220, *SE* = 0.078, *p* = .005, *p*_*corrected*_ = 0.035.

Neither a significant regression model was revealed for neutrophil deformability (M1: *F*(4, 131) = 1.086, *p* = .366, *R²* = 0.032, *Adj. R²* = 0.003. M2: *F*(5,130) = 0.883, *p* = .494, *Adj. R² =* -0.004, ∆*R²* = 0.001) nor for granulo-monocyte deformability (M1: *F*(4, 131) = 1.067, *p* = .376, *R²* = 0.032, *Adj. R²* = 0.002. M2: *F*(5,130) = 0.879, *p* = .497, *R²* = 0.033, *Adj. R² =* -0.004, ∆*R²* = 0.001), nor for erythrocyte deformability (M1: *F*(4, 131) = 1.465, *p* = .216, *R²* = 0.043, *Adj. R² =* 0.014. M2: *F*(5,130) = 1.246, *p* = .291, *R²* = 0.046, *Adj. R² =* 0.009, ∆*R²* = 0.003).

### Mediation of cortisol and cortisone (T1 and T2) on immune cell deformability

By applying a mediation analyses, we examined if long-term integrated glucocorticoids mediate the association between depressive severity at T1 and immune cell deformability at T2. Despite no association between depressive severity and neutrophil, granulo-monocyte as well as erythrocyte deformability, we exploratory conducted a mediation analyses to investigate the association between gluococorticoids and cell deformability of each cell type / cluster.

No significant mediation of hair cortisol levels (T1 + T2) regarding the association of depressive severity at T1 and cell deformability was exhibited. Neither was depressive severity associated with hair cortisol levels (ß = − 0.07, *p* = .543) nor were hair cortisol levels associated with neutrophil (ß = 0.08, *p* = .456), monocyte (ß = − 0.06, *p* = .569), lymphocyte (ß = 0.032, *p* = .806), granulo-monocyte (ß = 0.04, *p* = .671) or erythrocyte deformability (ß = − 0.04, *p* = .690).

Furthermore, no significant mediation of hair cortisone levels (T1 + T2) regarding the association of depressive severity at T1 and cell deformability was revealed. Neither was depressive severity associated with hair cortisone levels (ß = − 0.07, *p* = .539) nor were hair cortisone levels associated with neutrophil (ß = 0.14, *p* = .190), monocyte (ß = 0.03, *p* = .734), lymphocyte (ß = 0.059, *p* = .621), granulo-monocyte (ß = 0.12, *p* = .244) or erythrocyte deformability (ß = 0.00, *p* = .931). A detailed overview over all models can be taken from Table 2 and Supplementary Figs. 1 and 2.

## Discussion

### Summary of results

Depressive symptoms at T1 were positively associated with monocyte and lymphocyte deformability at T2, supporting our first hypothesis. However, results did partly not survive correction for multiple testing or the addition of confounders. Accumulated basal glucocorticoid levels at T1 and T2 neither mediated the association between depressive symptoms and cell deformability nor were they associated with cell deformability at T2, which is not in line with the second hypothesis. Notably, there was no association between depressive symptoms and cell size. Moreover, cell count of any cell type was neither associated with depressive symptoms nor with accumulated hair glucocorticoid levels.

### Integration of findings

To the authors knowledge, the present study is the first to examine the longitudinal relationship between depressive symptoms, glucocorticoid secretion and immune cell deformability in a population-based sample. The finding that elevated depressive symptomatology is associated with higher immune cell deformability one year later is supported by previously reported cross-sectional findings on the positive association between depressive disorder status, depressive symptoms and immune cell deformability [28]. Because these supporting findings originate from the same sample, it is important that further longitudinal as well as experimental studies replicate and extend these associations.

The observed significant positive associations between depressive symptoms at T1 and immune cell deformability at T2 for monocytes and lymphocytes in the entire sample and, as reported in Walther et al. (2022), for monocytes, neutrophils, and granulo-monocytes in the lifetime PDD and healthy control subsample, suggests these immune cells to be the most sensitive cells to react to depressive symptomatology with physical changes. These changes might be due to alterations in membrane-forming lipids, which have already been linked to DD in the central nervous system [67]. Moreover, since these cells are rich in glucocorticoid receptors [68–71], and HPA-axis alterations are considered a pathophysiological landmark of DD [14, 40], it would be reasonable to assume that changes in glucocorticoid secretion might also affect immune cell status and deformability. Indeed, previous research indicates that increased glucocorticoid levels led to increased immune cell counts due to cell demargination from the vessel walls, which was mediated by cellular softening [29]. However, the present study did not reveal any direct associations between accumulated glucocorticoid levels at T1 and T2 with cell deformability at T2 or with cell count at T2. Notably, we did not observe a positive association between levels of circulating immune cells and depressive symptoms in the present study (see Supplementary Table 2), although this was previously reported by another study for classificatory-categorical depression cases and depressive symptom severity [49]. In contrast to a previous report [43], no direct association between hair glucocorticoid levels and depressive symptoms emerged (see Table 4).

Our results suggest that accumulated long-term glucocorticoid concentrations over a one-year period do not affect immune cell structure and stability. Immune cells might respond only to short-term and synchronized increased glucocorticoid concentrations by reorganizing the cytoskeleton, which affects cell deformability directly as previous studies showed using dexamethasone administration [29, 72]. Hence, our assumption that elevated long-term glucocorticoid concentrations can cause increased permeation through the cell membrane affecting the lipidome and the cell structure leading to membrane destabilization and bending [20], and further to increased cell deformability could not be supported by our findings.

Compared to our case-control analyses in Walther et al. (2022), we did not detect increased neutrophil deformability with higher depression severity. Given the persistence of depressive symptoms in PDD over two years [3], associations with neutrophil cell deformability seem to be more detectable than in a snapshot regarding the presence of depressive symptoms within the last weeks. It is also likely that in PDD, the HPA axis shows hyperactivity with increased glucocorticoid output, as there is less depressive symptomatology but over a longer period of time than for example in MDD. In MDD, the HPA axis appears to enter exhaustion at some point, at which point a shift from increased to decreased levels of glucocorticoids are more likely [14, 46, 47, 73]. Thus, many reports also identify reduced hair cortisol levels or more complex patterns in MDD, especially in conjunction with experienced trauma [45, 74–76]. Therefore, future studies examining the relationship between DD or depressive symptoms, glucocorticoid output and immune cell deformability should aim to assess the time period since when individuals are suffering from depressive symptomatology as well as childhood maltreatment or trauma experience to better disentangle these seemingly complex associations.

Overall, the present study provides new insights into the pathophysiology of depressive symptoms, assuming more deformed immune cells, especially monocytes and lymphocytes over a small population-based sample. As long-term glucocorticoid output does not seem to be the underlying mechanism for the association of depressive symptoms and cell deformability, research needs to investigate whether further processes consistently linked to DD such as low-grade inflammation [15, 18, 77] or oxidative stress [30] might be additional factors contributing to cytoskeletal alterations and membrane destabilization [78, 79] resulting in higher immune cell deformability in DD or individuals suffering from depressive symptoms. This might be because, similar to increased glucocorticoid secretion, an inflammatory state leads to demargination of blood cells from the vessel walls into the circulation, with cell deformability being increased by the migration process [29]. Additionally, previous research indicates that an experimentally induced immune activation with lipopolysaccharide increased monocyte deformability [33], highlighting chronic low-grade inflammation in depressed individuals as potential agent for increased immune cell deformability. These assumptions are supported by findings of individuals with increased inflammatory signaling, such as healthy individuals after inhalation of lipopolysaccharide from E. coli, or individuals suffering from acute lung injury, viral respiratory infections, or Epstein-Barr virus infections, show increased deformability of neutrophils, monocytes, or lymphocytes [25]. Moreover, neutrophils of individuals in the acute phase of COVID-19 infection showed higher deformability, which was still observable also seven months after the acute infection symptoms [24], suggesting that long-term HPA-axis or inflammatory alterations may underlie immune cell deformability in DD.

It is important to mention that self-reported medication intake was not associated with immune cell deformability. As subjects were not drug naïve, we tested the association of different medication groups with cell deformability, such as psychopharmaceutical medication, antihypertensive drugs, and thyroid dysfunction medication. No association between any medication group and cell deformability was identified. Also, associations between depressive symptomatology and cell deformability cannot be traced back to cell size, as higher depression severity and cell size of immune cells were not associated with each other.

### Strengths and limitations

Some limitations must be taken into account when interpreting our results. The most important limitation is the single measurement of cell deformability at T2. To investigate the predictive value of depressive symptoms and accumulated glucocorticoid concentrations over a one-year period on cell deformability, we measured cell deformability as an outcome measure at T2. However, analyses of changes in cell deformability in relation to changes in depressive symptoms or glucocorticoid concentrations would also be important to investigate. Furthermore, with such a study design, the overall stability of cell deformability markers could be recorded. A related point is the single assessment of the diagnostic groups using a clinical interview at T2. Since we intended to capture group differences in cell deformability [28], the clinical diagnostics as well as the cell deformability measurement had to be performed at the same time point in order to avoid possible time effects. However, in a follow-up project, clinical diagnostics will also be performed at two time points together with cell deformability measurement. Nevertheless, our findings provide first evidence that depressive symptoms are related to immune cell deformability one year later, which are not mediated by long-term glucocorticoid secretion. Following on from this, however, it should be noted that other important systemic markers have not been investigated in the present study. Thus, it will be important in future studies to explore potential associations between inflammatory markers such as Interleukin-6 or C-reactive protein and immune cell deformability. In addition, the present sample of 136 participants might be underpowered to detect small to moderate effects, so only relatively large effects were probably identified in the present study further explaining the partly non-robust relationship between depressive symptoms and immune cell deformability. Finally, as gender differences and BMI partly play a role in this study, larger mixed samples or samples that focus only on women, men, or gender-diverse individuals need to be further investigated.

## Conclusions

Taken together, this study represents the first longitudinal investigation of the association between depressive symptoms, glucocorticoid concentrations, and peripheral immune cell deformability in a population-based sample. Our results suggest that higher levels of depressive symptoms are longitudinally associated with higher immune cell deformability, particularly in monocytes and lymphocytes. However, results have to be interpreted with caution due to lack of stability. Moreover, we could not confirm, that accumulated glucocorticoid concentrations over one year lead to cytoskeletal changes and cell membrane destabilization causing overall increased immune cell deformability. Increased immune cell deformability may either represent an adapted state of a regulatory immune response or a gradual loss of immune cell functionality, so that, for example, an adequate immune response is impaired or membrane function is compromised presenting a potentially underlying mechanism causing or maintaining depressive symptoms.

## Electronic supplementary material

Below is the link to the electronic supplementary material.

Supplementary Material 1

## Data Availability

The anonymized data and code will be made available to all interested parties upon request.
