# Supplementary material for: Longitudinal associations between depressive symptoms and cell deformability: do glucocorticoids play a role?
Source: Eur Arch Psychiatry Clin Neurosci. 2024 Sep 16;275(4):1075–85. doi: 10.1007/s00406-024-01902-z (PMC12148981; doi:10.1007/s00406-024-01902-z)
Supplement: Supplementary file 1 — Supplementary Material 1 [file 406_2024_1902_MOESM1_ESM.docx]

**Longitudinal Associations Between Depressive Symptoms and Cell Deformability: Do Glucocorticoids Play a Role?**

Julian Eder, M.Sc. M.A.^1*^; Martin Kräter, PhD^2,3^; Clemens Kirschbaum, PhD^1^; Wei Gao^1,4^; Magdalena Wekenborg^1, 5^; Marlene Penz^6^; Nicole Rothe^1^; Jochen Guck, PhD^2,3^; Lucas Daniel Wittwer, PhD ^3,7^; Andreas Walther, PhD^1,8*^

^1^ Biopsychology, Faculty of Psychology, TUD Dresden University of Technology, Dresden, Germany

^2^ Center for Molecular and Cellular Bioengineering, Biotechnology Center, TUD Dresden University of Technology, Dresden, Germany

^3^ Max Planck Institute for the Science of Light & Max-Planck-Zentrum für Physik und Medizin, Erlangen, Germany

^4^ School of Psychology, Nanjing Normal University, Nanjing, China

^5^ Else Kröner Fresenius Center of Digital Health, Faculty of Medicine and University Hospital Carl Gustav Carus, TUD Dresden University of Technology, Dresden, Germany

^6^ Institute of Psychology, Johannes Kepler Universität Linz, Linz, Austria

^7^Institut für numerische Mathematik und Optimierung, Technische Universität Freiberg, 09599 Freiberg, Germany

^8^ Clinical Psychology and Psychotherapy, University of Zurich, Zurich, Switzerland

*Corresponding authors

**Correspondence:** Dr. A. Walther: [a.walther@psychologie.uzh.ch](mailto:a.walther@psychologie.uzh.ch), Mobile Phone: +41783071816, Binzmühlestrasse 14, 8050, Zurich, Switzerland

**Supplementary Material**

**Supplementary Table 1.** Associations Between PHQ-9 (T1) and Cell Area According to the Entire Sample

| **Cell Area** | **Erythrocytes** | **Monocytes** | **Neutrophils** | **Lymphocytes** | **Granulo-Monocytes** |
| --- | --- | --- | --- | --- | --- |
| Pearson | -.030  *p* = .727 | .13  *p* = .133 | .067  *p* = .440 | -.071  *p* = .414 | .026  *p* = .763 |
| Kendall | -.051  *p* = .401 | .080  *p* = .183 | .060  *p* = .324 | -.027  *p* = .651 | .041  *p* = 494 |
| Partial Correlation | -.047  *p* = .594 | .135  *p* = .123 | .056  *p* = .524 | -.093  *p* = .288 | .018  *p* = .837 |

*Note*. Pearson and Kendall tests were calculated depending on normality and scale of measurement (*n* = 136). Partial correlations include age, gender, BMI and psychopharmaceutical intake as confounders. All correlations were calculated two-tailed.

**Supplementary Table 2.** *Associations Between Depressive Symptoms and Cell Count According to the Entire Sample*

|  | **Erythrocytes** | **Monocytes** | **Neutrophils** | **Lymphocytes** | **Granulo-Monocytes** |
| --- | --- | --- | --- | --- | --- |
| **Entire Sample (*n* = 136)** |  |  |  |  |  |
| Pearson | .031  *p* = .717 | -.030  *p* = 0.727 | .022  *p* = .797 | -.062  *p* = .476 | -.001  *p* = .987 |
| Kendall | .022  *p* = .712 | .045  *p* = .462 | .013  *p* = .824 | -.089  *p* = .134 | -.004  *p* = .953 |
| Partial correlation | 0.036  *p* = .712 | -.054  *p* = .538 | .027  *p* = .755 | -.040  *p* = .649 | -.008  *p* = .924 |

*Note*. Pearson and Kendall tests were calculated depending on normality and scale of measurement (*n* = 136). Partial correlations include age, gender, BMI and psychopharmaceutical intake as confounders. All correlations were calculated two-tailed.

**Supplementary Table 3**. *Associations Between Accumulated Hair Cortisol Levels and Cell Count According to the Entire Sample*

|  | **Erythrocytes** | **Monocytes** | **Neutrophils** | **Lymphocytes** | **Granulo-Monocytes** |
| --- | --- | --- | --- | --- | --- |
| **Entire Sample (*n* = 113)** |  |  |  |  |  |
| Pearson | .097  *p* = .307 | -.042  *p* = .661 | .084  *p* = .378 | .070  *p* = .461 | .097  *p* = .308 |
| Kendall | .062  *p* = .335 | .031  *p* = .645 | .047  *p* = .457 | .100  *p* = .115 | .074  *p* = .245 |

*Note*. Pearson and Kendall tests were calculated depending on normality and scale of measurement (*n* = 113). All correlations were calculated two-tailed.

**Supplementary Table 4.** *Associations Between Accumulated Hair Cortisone Levels and Cell Count According to the Entire Sample*

|  | **Erythrocytes** | **Monocytes** | **Neutrophils** | **Lymphocytes** | **Granulo-Monocytes** |
| --- | --- | --- | --- | --- | --- |
| **Entire Sample (*n* = 115)** |  |  |  |  |  |
| Pearson | .080  *p* = .396 | .000  *p* = .999 | .005  *p* = .955 | .134  *p* = .227 | .029  *p* = .758 |
| Kendall | .044  *p* = .491 | .014  *p* = .828 | -.013  *p* = .835 | .119  *p* = .061 | .012  *p* = .757 |

*Note*. Pearson and Kendall tests were calculated depending on normality and scale of measurement (*n* = 115). All correlations were calculated two-tailed.

**Supplementary Table 5***. Results of Hierarchical Regression Analyses for Depressive Symptoms and Lymphocyte Cell Deformability*

| Predictor | ß | *b* | 95% CI | *SE* | *t* | *p* |
| --- | --- | --- | --- | --- | --- | --- |
| Model 1 |  |  |  |  |  |  |
| Intercept | .022 | 0.0255 | [0.023, 0.028] | 0.160 | 0.137 | .891 |
| Age T2 | .030 | 0.00001 | [-0.00, 0.000] | 0.087 | 0.349 | .727 |
| Gender T2 (ref == female) | -.018 | -0.00003 | [-0.001 0.001] | 0.194 | -0.095 | .925 |
| BMI T2 | **.336**** | 0.0001 | [0.0001,0.0002] | 0.103 | 3.271 | **.001** |
| Psychopharmaceutical Intake T2 (ref == intake) | -.050 | -0.00009 | [-0.001, 0.001] | 0.218 | -0.229 | .819 |
| Model 2 |  |  |  |  |  |  |
| Intercept | .061 | 0.0252 | [0.023, 0.027] | 0.155 | 0.392 | .696 |
| Age T2 | .042 | 0.00001 | [-0.000, 0.000] | 0.084 | 0.499 | .619 |
| Gender T2 (ref == female) | -.029 | -0.0001 | [-0.001, 0.001] | 0.190 | -0.154 | .878 |
| BMI T2 | **.323**** | 0.0001 | [0.000, 0.000] | 0.098 | 3.282 | **.001** |
| Psychopharmaceutical Intake T2 (ref == intake) | -.232 | -0.0004 | [-0.001, 0.000] | 0.229 | -1.011 | .314 |
| PHQ-9 T1 | **.172*** | 0.00005 | [0.000, 0.000] | 0.081 | 2.115 | **.036** |

*Note.* 95% CI is based on 1000 bootstrap replicates. Continuous variables are mean-centered and scaled by 1 *SD*. Standard errors and t-values are calculated with HC4 for standardized coefficients. Model fit 1: *F*(4, 131) = 4.495, *p* = .002, *R²* = .121, *Adj. R²* = .094. Model fit 2: *F*(5,130) = 4.428, *p* = <.001, *R²* = .146, *Adj.* R² *=* .113, ∆*R²* = .025; Model 1 vs. model 2: *F*(1,130) = 3.780, *p* = .054. Normal distribution of residuals is not met (M1: *W* = .979, *p* = .038; M2: *W* = .981, *p* = .0497). ***p* < .01; **p* < .05.

**Supplementary Table 6***. Results of Hierarchical Regression Analyses for Depressive Symptoms and Monocyte Cell Deformability*

| Predictor | ß | *b* | 95% CI | *SE* | *t* | *p* |
| --- | --- | --- | --- | --- | --- | --- |
| Model 1 |  |  |  |  |  |  |
| Intercept | -.322 | 0.05103 | [0.044, 0.056] | 0.165 | -1.944 | .054 |
| Age T2 | -.028 | -0.00002 | [-0.000 0.000] | 0.091 | -0.311 | .756 |
| Gender T2 (ref == female) | **.393*** | 0.00258 | [0.000, 0.005] | 0.191 | 2.056 | **.042** |
| BMI T2 | .106 | 0.00014 | [-0.000, 0.000] | 0.088 | 1.210 | .228 |
| Psychopharmaceutical Intake T2 (ref == intake) | .192 | 0.00126 | [-0.002 0.004] | 0.245 | 0.785 | .434 |
| Model 2 |  |  |  |  |  |  |
| Intercept | -.273 | 0.04972 | [0.043, 0.056] | 0.153 | -1.786 | .076 |
| Age T2 | -.014 | -0.00001 | [-0.000, 0.00] | 0.090 | -0.152 | .879 |
| Gender T2 (ref == female) | **.380*** | 0.00249 | [0.000, 0.005] | 0.183 | 2.071 | **.040** |
| BMI T2 | .091 | 0.00012 | [-0.000, 0.000] | 0.088 | 1.025 | .307 |
| Psychopharmaceutical Intake T2 (ref == intake) | -.036 | -0.00024 | [-0.004, 0.003] | 0.246 | -0.147 | .884 |
| PHQ-9 T1 | **.216**** | 0.00020 | [0.00004, 0.0004] | 0.078 | 2.761 | **.007** |

Note. 95% CI is based on 1000 bootstrap replicates. Continuous variables are mean-centered and scaled by 1 *SD*. Standard errors and t-values are calculated with HC4 for standardized coefficients. Model fit 1: F(4, 131) = 1.325, p = .264, R² = .039, *Adj.* R² = .010. Model fit 2: *F*(5,130) = 2.201, *p* = 0.058 , R² = 0.078, *Adj. R² =* 0.043 , ∆R² = 0.039. Model 1 vs. model 2: *F*(1,130) = 5.522, *p* = .020. All assumptions are met. ***p* < .01; **p* < .05.

**Supplementary Table 7.** *Results of Hierarchical Regression Analyses for Depressive Symptoms and Neutrophil Cell Deformability*

| Predictor | ß | *b* | Bca 95% CI | *SE* | *t* | *p* |
| --- | --- | --- | --- | --- | --- | --- |
| Step 1 |  |  |  |  |  |  |
| Intercept | .046 | 0.061 | [0.056, 0.065] | 0.148 | 0.310 | .757 |
| Age T2 | .083 | 0.00003 | [-0.000, 0.000] | 0.101 | 0.823 | .412 |
| Gender T2 (ref == female) | -.138 | -0.001 | [-0.002, 0.001] | 0.190 | -0.723 | .471 |
| BMI T2 | .027 | 0.00002 | [-0.000, 0.000] | 0.113 | 0.243 | .808 |
| Psychopharmaceutical Intake T2 (ref == intake) | .329 | 0.001 | [-0.001, 0.003] | 0.225 | 1.459 | .147 |
| Step 2 |  |  |  |  |  |  |
| Intercept | .052 | 0.06055 | [0.056, 0.065] | 0.150 | 0.349 | .728 |
| Age T2 | .085 | 0.00003 | [-0.000, 0.000] | 0.101 | 0.838 | .404 |
| Gender T2 (ref == female) | -.140 | -0.00060 | [-0.002, 0.001] | 0.191 | -0.730 | .466 |
| BMI T2 | .025 | 0.00002 | [-0.000, 0.000] | 0.115 | 0.220 | .826 |
| Psychopharmaceutical Intake T2 (ref == intake) | .297 | 0.00128 | [-0.001, 0.003] | 0.236 | 1.260 | .210 |
| PHQ-9 T1 | .030 | 0.00002 | [-0.000, 0.000] | 0.087 | 0.343 | .732 |

Note. 95% CI is based on 1000 bootstrap replicates. Continuous variables are mean-centered and scaled by 1 *SD*. Standard errors and t-values are calculated with HC4 for standardized coefficients. Model fit 1: *F*(4, 131) = 1.086, *p* = .366, *R²* = 0.032, *Adj. R²* = 0.003. Model fit 2: *F*(5,130) = 0.883, *p* = .494, *R²* = 0.033, *Adj. R² =* -0.004, ∆R² = .0008; Model 1 vs. model 2; *F*(1,130) = 0.102, *p* = .751.

**Supplementary Table 8.** *Results of Hierarchical Regression Analyses for Depressive Symptoms and Granulo-Monocyte Cell Deformability*

| Predictor | ß | *b* | 95% CI | *SE* | *t* | *p* |
| --- | --- | --- | --- | --- | --- | --- |
| Model 1 |  |  |  |  |  |  |
| Intercept | .010 | 0.06019 | [0.056, 0.064] | 0.148 | 0.068 | .946 |
| Age T2 | .075 | 0.00003 | [-0.000, 0.000] | 0.096 | 0.783 | .435 |
| Gender T2 (ref == female) | -.089 | -0.00039 | [-0.002, 0.001] | 0.188 | -0.473 | .637 |
| BMI T2 | .052 | 0.00005 | [-0.000, 0.000] | 0.106 | 0.495 | .621 |
| Psychopharmaceutical Intake T2 (ref == intake) | .328 | 0.00142 | [-0.0003,0.003] | 0.222 | 1.475 | .143 |
| Model 2 |  |  |  |  |  |  |
| Intercept | .018 | 0.06004 | [0.056, 0.06] | 0.149 | 0.123 | .902 |
| Age T2 | .078 | 0.00003 | [-0.000, 0.000] | 0.096 | 0.807 | .421 |
| Gender T2 (ref == female) | -.091 | -0.00040 | [-0.002, 0.001] | 0.188 | -0.485 | .629 |
| BMI T2 | .050 | 0.00004 | [-0.000 0.0] | 0.107 | 0.462 | .645 |
| Psychopharmaceutical Intake T2 (ref == intake) | .289 | 0.00125 | [-0.001 0.003] | 0.235 | 1.230 | .221 |
| PHQ-9 T1 | .037 | 0.00002 | [-0.000, 0.000] | 0.086 | 0.432 | .666 |

Note. 95% CI is based on 1000 bootstrap replicates. Continuous variables are mean-centered and scaled by 1 *SD*. Standard errors and t-values are calculated with HC4 for standardized coefficients. Model fit 1: *F*(4, 131) = 1.067, *p* = .376 , R² = 0.032, *Adj.* R² = 0.002. Model fit 2: *F*(5,130) = 0.879, *p* = 0.497, R² = 0.033, *Adj. R² =* -0.004, ∆R² = 0.001. Model 1 vs. model 2; *F*(1,130) = 0.156, *p* = .694. Normal distribution of residuals is not met (M1: *W* = 0.955, *p* < .001; M2: *W* = 0.955, *p* < .001).

**Supplementary Table 9.** *Results of Hierarchical Regression Analyses for Depressive Symptoms and Single Red Blood Cell Deformability*

| Predictor | ß | *b* | 95% CI | *SE* | *t* | *p* |
| --- | --- | --- | --- | --- | --- | --- |
| Step 1 |  |  |  |  |  |  |
| Intercept | .167 | 0.358 | [0.340, 0.381] | 0.170 | 0.982 | .328 |
| Age T2 | -.033 | -0.00005 | [-0.0003, 0.000] | 0.099 | -0.334 | .739 |
| Gender T2 (ref == female) | -.210 | -0.003 | [-0.009, 0.003] | 0.193 | -1.092 | .277 |
| BMI T2 | -.189 | -0.0006 | [-0.001, -0.000] | 0.100 | -1.881 | .062 |
| Psychopharmaceutical Intake T2 (ref == intake) | -.074 | -0.0012 | [-0.009, 0.007] | 0.273 | -0.271 | .787 |
| Step 2 |  |  |  |  |  |  |
| Intercept | .180 | 0.358 | [0.336, 0.379] | 0.168 | 1.074 | .285 |
| Age T2 | -.029 | -0.00004 | [-0.0003, 0.000] | 0.099 | -0.294 | .770 |
| Gender T2 (ref == female) | -.214 | -0.003 | [-0.009, 0.002] | 0.192 | -1.116 | .266 |
| BMI T2 | -.193 | -0.001 | [-0.001, -0.000] | 0.102 | -1.891 | .061 |
| Psychopharmaceutical Intake T2 (ref == intake) | -.137 | -0.002 | [-0.011, 0.008] | 0.303 | -0.450 | .653 |
| PHQ-9 T1 | .059 | 0.0001 | [-0.000, 0.001] | 0.097 | 0.609 | .544 |

Note. 95% CI is based on 1000 bootstrap replicates. Continuous variables are mean-centered and scaled by 1 SD. Standard errors and t-values are calculated with HC4 for standardized coefficients. Model fit 1: *F*(4, 131) = 1.465, *p* = .217 , *R²* = 0.043, *Adj. R² =* 0.014. Model fit 2: *F*(5,130) = 1.246, *p* = .291, *R²* = 0.046, *Adj. R² =* 0.009, *∆R²* = 0.003. Model 1 vs. model 2: *F*(1,130) = 0.398, *p* = .529. All assumptions are met.

**Supplementary Text 1**

Drug intake, measured by the subject questionnaire, was analysed by forming six groups, which are: no medication (*n* = 76), psychopharmaceutical medication (*n* = 14), antihypertensive drugs (*n* = 16), thyroid dysfunction medication (*n* = 16), others (*n* = 4) and an intake combination group of the preceding categories (*n* = 10). ANOVA revealed no significant difference between the medication intake groups and cell deformation for each cell type [red blood cells (*F*_(5, 130)_ = 1.163, *p* = .331), monocytes (*F*_(5, 130)_ = 1.112 , *p* = .357), neutrophils (*F*_(5, 130)_ = .722 , *p* = .608), lymphocytes (*F*_(5, 130)_ = .935, *p* = .461), granulo-monocytes (*F*_(5, 130)_ = .897, *p* = .486)].

**Supplementary Figure 1.** Mediating Effect of Accumulated Hair Cortisol Levels (T1 +T2) Regarding the Association of Depressive Symptoms (T1) and Cell Deformability (T2)

| 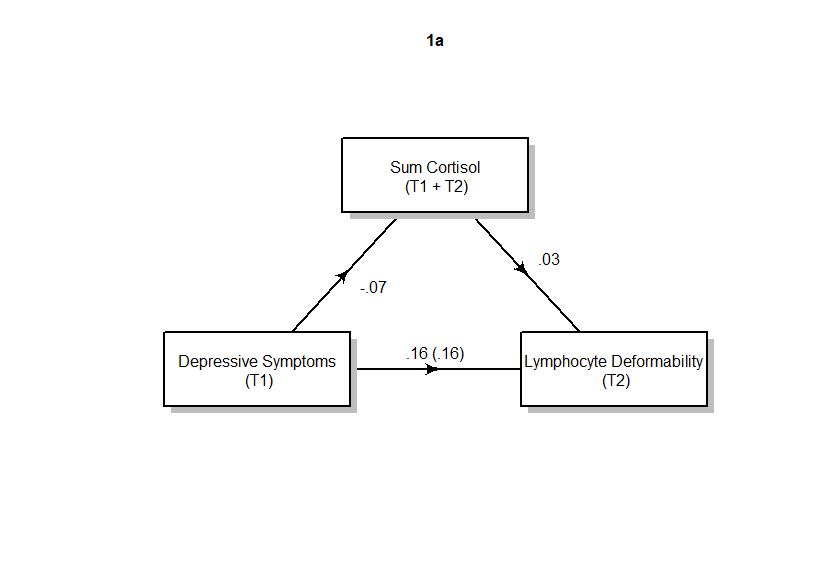 | | 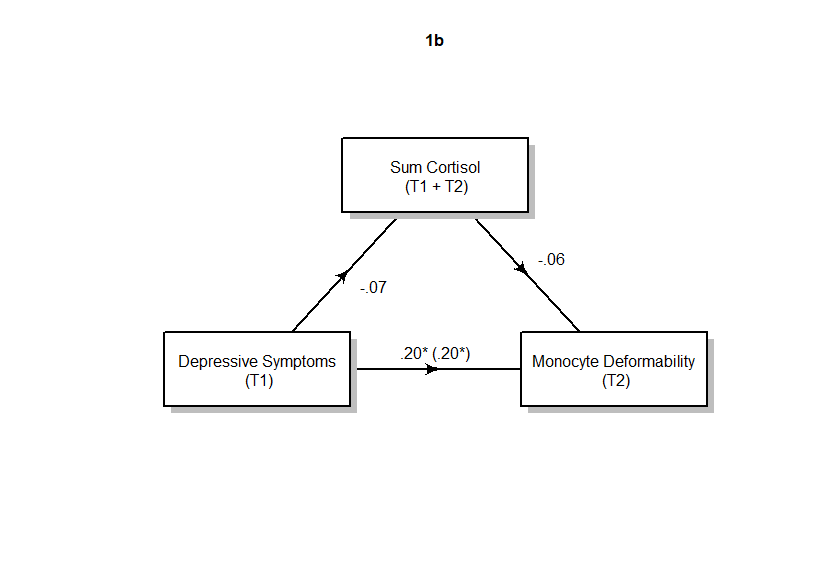 | |
| --- | --- | --- | --- |
| 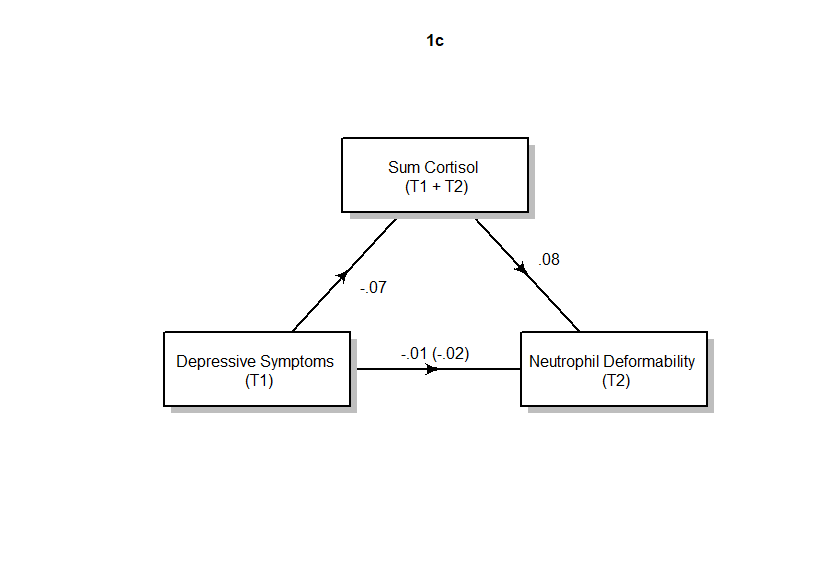 | 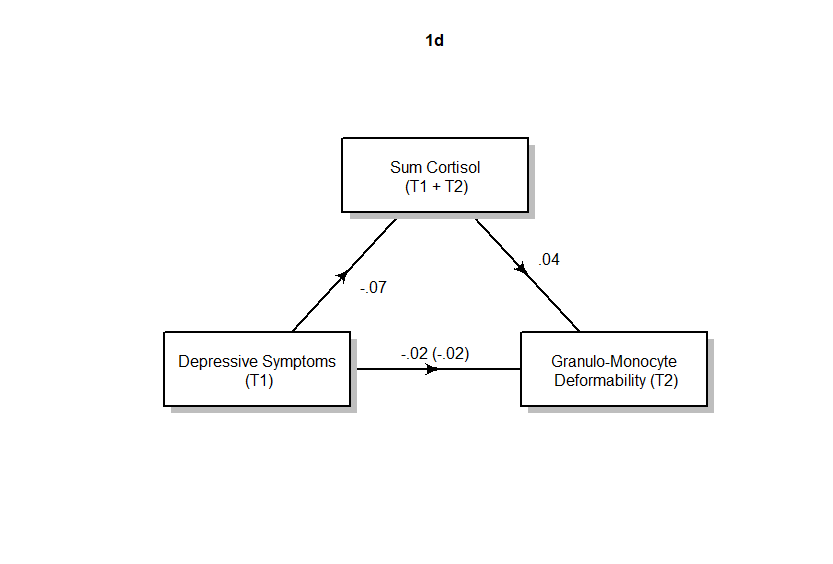 | | 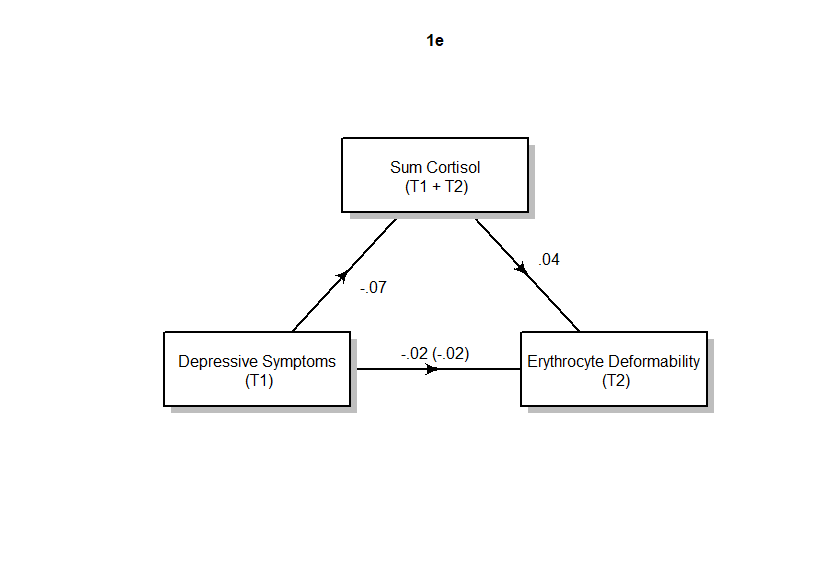 |

*Note*. Independent variable = PHQ-9 (T1); Dependent variable = Cell deformability (T2). Mediators: accumulated hair cortisol concentrations (T1+T2). Age, gender, BMI and psychopharmaceutical intake are taken into account as confounders. Sample size differ for the displayed c-path (n = 113) compared to hierarchical regression analyses *n* = 136. * *p* < .05.

**Supplementary Figure 2.** Mediating Effect of Accumulated Hair Cortisone Levels (T1 +T2) Regarding the Association of Depressive Symptoms (T1) and Cell Deformability (T2)

| 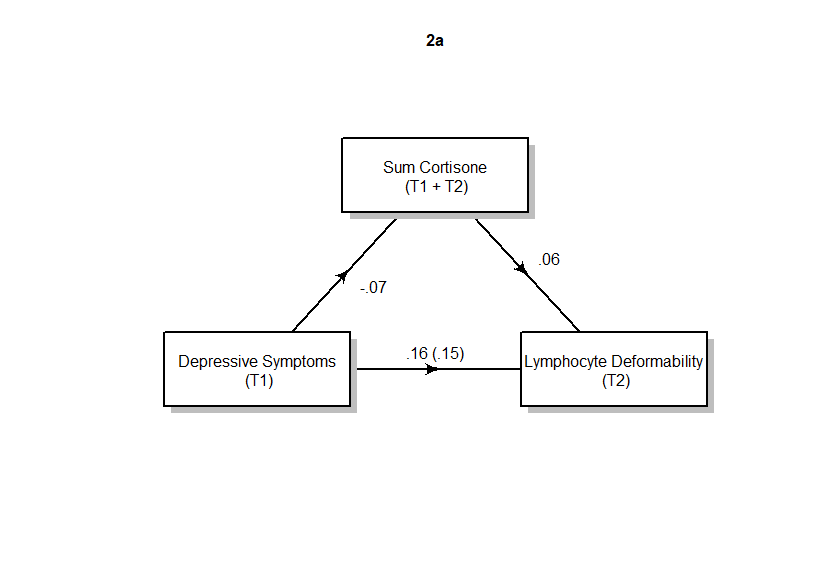 | | 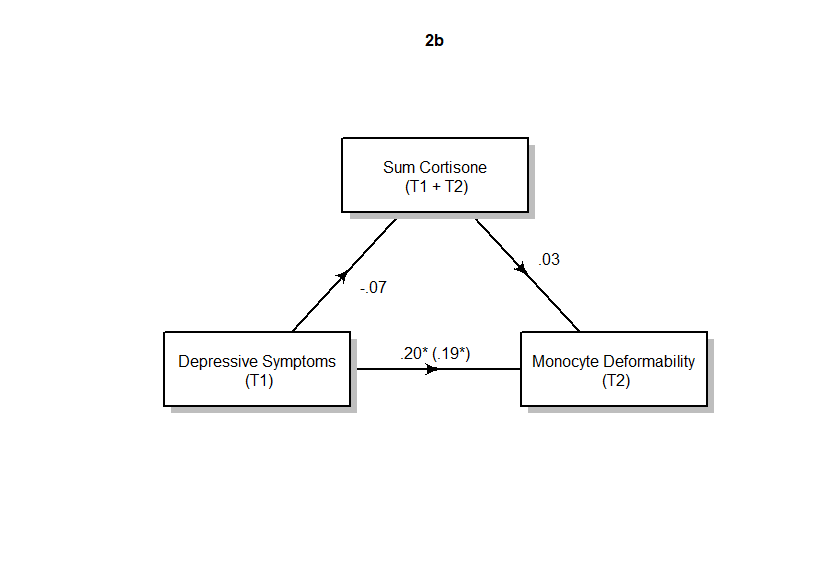 | |
| --- | --- | --- | --- |
| 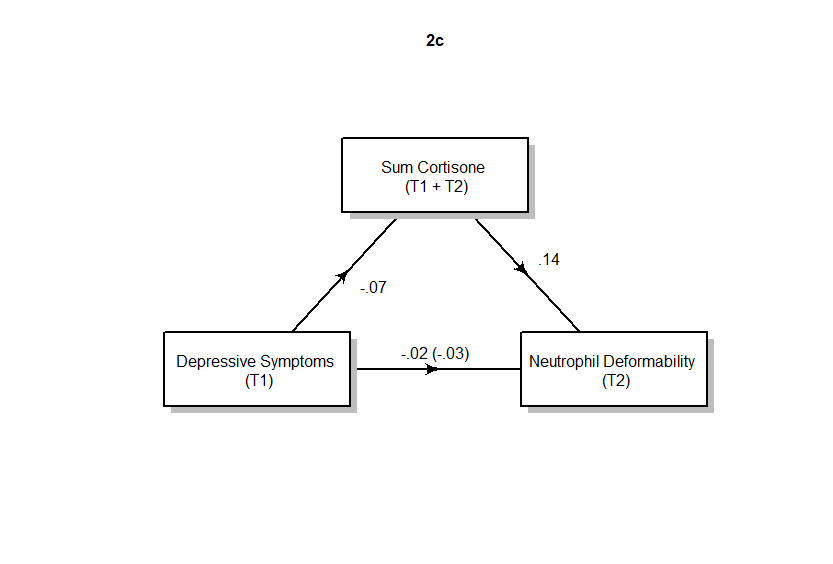 | 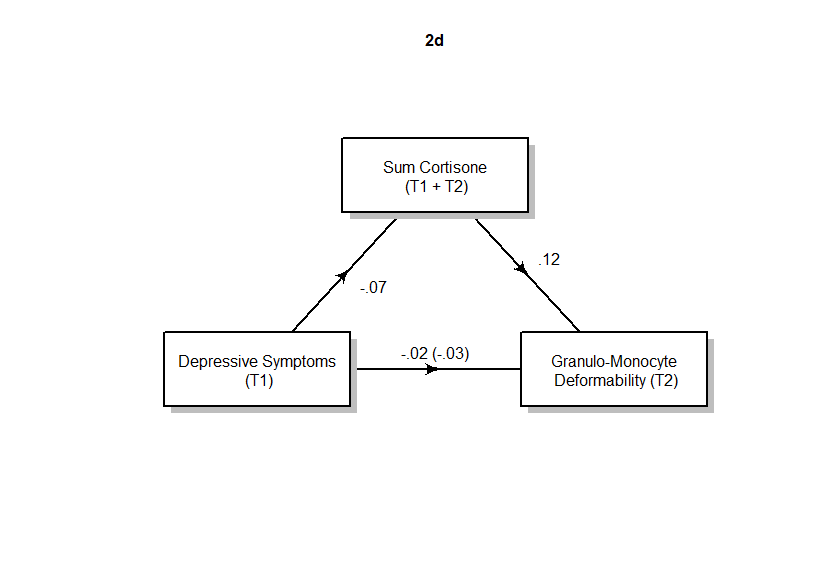 | | 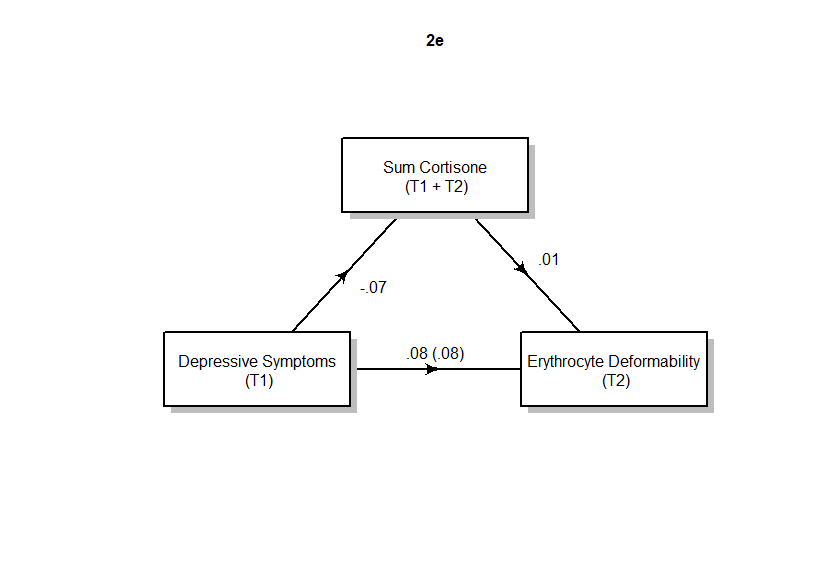 |

*Note*. Independent variable = PHQ-9 (T1); Dependent variable = Cell deformability (T2). Mediators: accumulated hair cortisone concentrations (T1+T2). Age, gender, BMI and psychopharmaceutical intake are taken into account as confounders. Sample size differ for the displayed c-path (n = 115) compared to hierarchical regression analyses *n* = 136. * *p* < .05.
